# TNF-*α* Downregulation Modifies Insulin Receptor Substrate 1 (IRS-1) in Metabolic Signaling of Diabetic Insulin-Resistant Hepatocytes

**DOI:** 10.1155/2019/3560819

**Published:** 2019-02-04

**Authors:** Iraj Alipourfard, Nelly Datukishvili, Davit Mikeladze

**Affiliations:** Institute of Chemical Biology, School of Natural Sciences and Engineering, Ilia State University, Tbilisi 0162, Georgia

## Abstract

One of the major mechanisms of hyperglycemia in type 2 diabetes is insulin resistance (IR) which can induce free fatty acids like palmitate. In hepatic cell, as an insulin target tissue, insulin resistance can be stimulated by inflammatory cytokine TNF-*α*. The interaction of intracellular TNF-*α* signal with the insulin signaling pathway is not well identified. Hence, we aimed to investigate the effect of TNF-*α* elimination on the diabetic model of palmitate-induced insulin-resistant hepatocytes (HepG2). The changes of phosphorylation rate in IRS-1 protein are determined to know the effect of TNF-*α* on this key protein of the insulin signaling pathway. HepG2 cells were treated with 0.5 Mm palmitate, and TNF-*α* gene knockdown was performed by shRNA-mediated technique. Western blot analysis was used to evaluate the phosphorylated activity of the insulin signaling pathway. Palmitate-induced IR could increase TNF-*α* protein expression 1.2-, 2.78-, and 2.25-fold compared to the control cells at times of 8 h, 16 h, and 24 h, respectively. TNF-*α* expression in downregulated cells transfected with shRNA-TNF-*α* is approximately 47.0% of normal cells and 49.0% in the case of scrambled cells. IRS-1 phosphorylation in TNF-*α*-downregulated and stimulated cells with 100 nM insulin, after treatment and in the absence of palmitate, was 45% and 29% higher than the normal cells. These data support the evidence that TNF-*α* downregulation strategy contributes to the improvement of IRS-1 phosphorylation after insulin stimulation and insulin response in HepG2 liver cells.

## 1. Introduction

Millions of people around the world have been diagnosed with type 2 diabetes, and many more remain undiagnosed. It has been considered as epidemic-like proportion since it is likely to be more than double by 2030 [1] and type 2 accounts for 90% of all cases of diabetes encompassing both developed and developing nations. Hepatic insulin resistance (IR) is thought to be the main factor in the development of fasting hyperglycemia [2]. Hepatic gluconeogenesis alone contributes 50-60% of HGP (hepatic glucose production) and is the primary reason for the increase of fasting glucose levels in individuals with type 2 diabetes [3]. The infusion of FFA (free fatty acids) such as PA (palmitic acid) in normal and obese insulin-resistant individuals enhances HGP by the stimulation of gluconeogenesis [4]. The mechanisms by which FFA induces insulin resistance in both humans and rodents have been elucidated. In the liver, increased levels of DAG (diacylglycerol) resulting from FFA plasma elevation reduce tyrosine phosphorylation of IRS (insulin receptor substrate). The principal role has been demonstrated for IRS-1 and IRS-2 as a link of cell surface receptors to the intracellular signaling cascades [5]. Increased activation of IRS stimulates glycogen synthase and glycogen synthesis and subsequently increased glucose output. Similar to FFA, inflammatory cytokines like TNF-*α*, IL-6, and IL1*β* can also impair the insulin signaling pathway leading to insulin-resistant metabolic conditions [6, 7]. The role of TNF-*α* in insulin resistance of adipocytes and in the stimulation of lipolysis indicates hyperlipidemia and peripheral insulin resistance. It has been supported by the fact that in obesity and high-fat diet, removal of TNF-*α* function improves insulin sensitivity and glucose homeostasis in obese mice [8–11]. In addition, an acute TNF-*α* infusion in healthy humans leads to insulin resistance through impaired insulin signaling and decreased glucose uptake [7, 11]. The binding of TNF-*α* to the cell surface receptor leads to the activation of two major transcription factors: c-Jun and nuclear factor-*κβ* (NF-*κβ*), which subsequently results in the activation of genes involved in the inflammatory process, stress responses, and cell growth and development [6, 7, 9]. The previous studies have been shown that saturated fatty acids especially palmitate can induce TNF-*α* expression in insulin-resistant obese muscle cells [12–14]. However, the attribution of TNF-*α* expression in pathogenesis of palmitate-induced insulin resistance and inflammation in liver cells is poorly described. The current study is aimed at investigating the effect of TNF-*α* elimination on the palmitate-induced insulin resistance. It is an insight into the regulation of the hepatic insulin signaling pathway and glucose uptake through IRS. We determined the beneficiary phosphorylation of this key protein in TNF-*α* knockdown and control hepatic (HepG2) cells under the presence and absence of PA. It has been purposed to demonstrate the novel potential background for eliminated expression of the inflammatory factor TNF-*α* in the improvement of hepatic diabetic cells.

## 2. Material and Methods

### 2.1. Fatty Acid (Palmitate) Preparation

Palmitate was prepared according to the protein absorption method [7]. To increase the solubility of PA, it should be conjugated to BSA with the equal ratio. Firstly, PA was prepared in 0.1 mM NaOH by heating up to 70°C; then PA shock solution was added dropwise to prewarmed 10% *w*/*w* endotoxin/fatty acid-free BSA to make a 50 mM working stoke and incubated in a water bath. The conjugated PA solution was sterile filtered and kept in -20°C.

### 2.2. Hepatic Cell Culture and Treatments

Hepatocellular carcinoma cells (HepG2 cells) possess the same bioactivity as normal hepatic cells. These cells are valuable for investigating liver-associated functions, and they are stable during many passages. The HepG2 cell line was purchased and cultured in high-glucose Dulbecco's Modified Eagle's Medium (DMEM) (Gibco, Berlin, Germany). It has been supplemented with 10% *v*/*v* heat-inactivated fetal bovine serum (FBS) and 1% *v*/*v* penicillin/streptomycin antibiotic solution at 37°C under 5% CO_2_ atmosphere. For mRNA or protein analysis, HepG2 cells were cultured for 24 h in DMEM supplemented with 1% FBS and the presence or absence of PA (25 mM). For PA treatment, the PA-free BSA samples were incubated at 37°C for 2 hours and used for the treatment of HepG2-cultured cells.

### 2.3. shRNA Transfection Targeting TNF-*α* Gene

In order to knock down TNF-*α* gene, shRNA-mediated technique was performed [15] by using shRNA lentiviral particles (Santa Cruz Biotechnology Inc., Heidelberg, Germany), which were designed to suppress the production of TNF-*α* in liver cells. HepG2 cells were transduced with shRNA lentiviral particles and with noneffective scrambled shRNA sequences as the control (scrambled control). The cells at density of 3 × 10^5^ cells per dish were seeded onto 35 mm dishes. After one day of seeding, 200 ml of lentiviral particles in 2 ml DMEM medium including 10% FBS was added to the cultures and then incubated for 24 h in 5% CO_2_ at 37°C. The cells successfully infected by lentiviral particles were selected using 3 mg/ml puromycin in the presence of 10% FBS for 48 h. The processed cells were then harvested and applied to Western blot analysis. A sample checking was done to determine the efficiency of the used lentiviral particles for suppressing TNF-*α* gene expression.

### 2.4. Western Blot Analysis

To confirm the downregulation of the TNF-*α* protein level, Western blot method was used [6, 7, 15]. Also, to determine the positive phosphorylation (which leads to increase of activation) of IRS-1 as a key element in the insulin signaling pathway, Western blotting was performed semiquantitatively in the presence and absence of palmitate. In addition, to eliminate the errors due to unequal loaded amounts of protein samples, the structural protein beta-actin was used as housekeeping gene. HepG2 cell lysate was prepared by homogenization in modified RIPA buffer (50 Mm Tris-HCl, pH 7.4, 1% Triton X-100, 0.2% sodium deoxycholate, 0.2% SDS, 1 mM Na-EDTA, 1 mM PMSF) and supplemented with protease inhibitor cocktail (Roche, Mannheim, Germany). For the detection of phosphoprotein, a buffer consisting of 50 mM HEPES pH 7.5, 150 mM NaCl, 100 mM NaF, 10 mM EDTA, 10 mM Na_4_P_2_O_7_, 2 mM NaVO_4_, and protease inhibitor cocktail was used. Protein concentration was determined using Bradford's method [16]. 20-30 mg of total protein was fractionated by SDS-PAGE. The gel was then transferred onto a PVDF membrane (Millipore, Schwalbach, Germany), blocked in blocking buffer overnight (5% skimmed milk in TBST buffer), and incubated for 1 h with primary antibodies diluted in TBST containing 1% BSA. Primary antibodies used were as follows: TNF-*α*, p-IRS-1 (Tyr632) (Santa Cruz Biotechnology Inc., USA), and *β*-actin (Abcam, Cambridge, MA, USA). The membrane was then incubated with secondary antibody conjugated to HRP (Santa Cruz Biotechnology Inc., USA) for 1 hour, and detection was performed using ECL reagents (Amersham Pharmacia Corp., Piscataway, NJ, USA). Films were scanned, and protein bands were quantified using the TotalLab software. The density of different protein bands measured and the density of TNF-*α* band of each sample were divided to the density of *β*-actin on the same sample to remove loading-related errors. Each experiment was performed at least three times.

### 2.5. Statistical Assays

The statistical analyses were performed by SPSS 19.0 (SPSS, Chicago, IL) software. All normally distributed continuous variables were expressed as mean ± SD. Each experiment was repeated at least three times. The comparison between all continuous variables was performed by using one-way analysis of variance (ANOVA). If there were statistically significant differences, then Tukey's post hoc test was applied. The values of *p* < 0.05 were considered statistically significant.

## 3. Results

### 3.1. The Expression of TNF-*α* Was Amplified by Palmitate in HepG2 Insulin Resistance Model Cell

**Figure 1:**
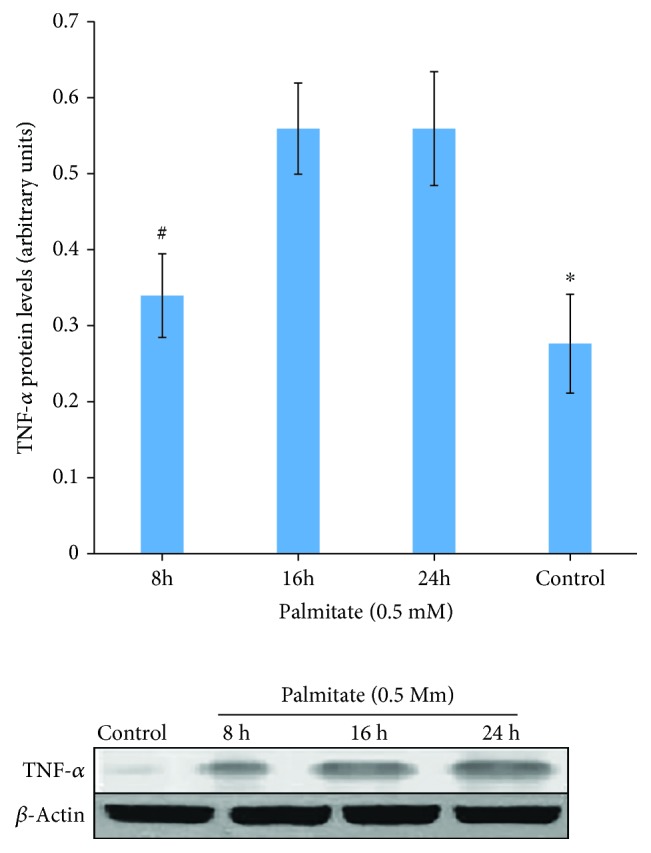
Effect of palmitate on TNF-*α* protein expression in HepG2 cells before knockdown. Time course of TNF-*α* protein expression in the presence of 0.5 mM palmitate. Western blot analysis was performed using antibodies against TNF-*α* and *β*-actin (internal control). The level of TNF-*α* protein was normalized by *β*-actin protein. ^∗^*p* < 0.01 vs. all other groups. ^#^*p* < 0.05 vs. 16 h and 24 h.

The treatment by 0.5 mM palmitate within different time courses augmented (*p* < 0.05) the expression levels of TNF-*α* in HepG2 cells (Figure 1). These increments in TNF-*α* expression at times 8 h, 16 h, and 24 h were approximately 1.2-, 2.78-, and 2.25-fold compared to the control cells, respectively. Based on the statistical analysis, an obvious significant difference in the TNF-*α* protein level between treatment time of 8 h and the other times was found. However, there was no significant difference between the times of 16 h and 24 h. Hence, the treatment time 16 was prioritized to pursue the following palmitate treatments.

### 3.2. Alleviation of TNF-*α* Expression in Hepatocytes

**Figure 2:**
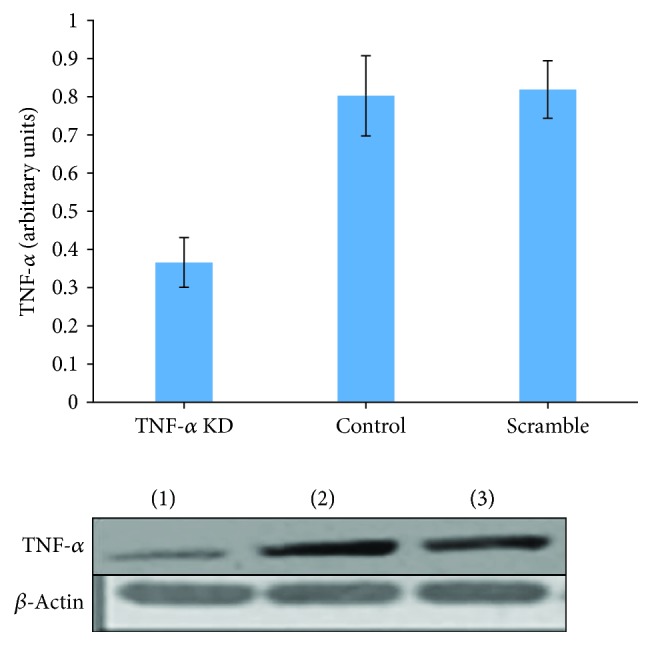
Reduction of TNF-*α* expression in HepG2 cells following transfection with the lentiviral particle method: (1) shRNA-TNF-*α* plasmid, (2) plasmid lacks shRNA-TNF-*α*, and (3) nonsense plasmid using HepG2 cells transfected with *μ*20 g of each plasmid 1-3. 48 hours later, Western blot was performed for TNF-*α* and *β*-actin as internal control. The amounts of TNF-*α* protein normalized by considering the amounts of *β*-actin protein. The data has been achieved of three independent experiments and shown as mean ± SD; *p* < 0.05.

A gene silencing approach directed to TNF-*α* (shRNA-TNF-*α*) was used to knock down TNF-*α* gene in hepatocytes. Subsequently, the rate of TNF-*α* protein expression was analyzed by the Western blot method (Figure 2). HepG2 cells were transfected with plasmids containing shRNA-TNF-*α* (TNF-*α* knockdown cells), plasmids lacking shRNA-TNF-*α* (control cells), and plasmids containing sh-RNA which interferes with none of the intracellular mRNA (scrambled cells). The transfection procedures were done by a related commercial method. The concentration of TNF-*α* produced by hepatocytes was quantitated [16], and equal amounts of each sample supernatants were loaded to perform Western blot analysis. The results of Western blotting have demonstrated that the TNF-*α* expression in knockdown cells transfected with shRNA-TNF-*α* is approximately 47.0% of control cells. In the case of scrambled cells, it is 49.0% compared to the control cells.

### 3.3. Downregulation of TNF-*α* Encouraged the Insulin Signaling Pathway in PA-Induced Insulin Resistance HepG2 Cells

**Figure 3:**
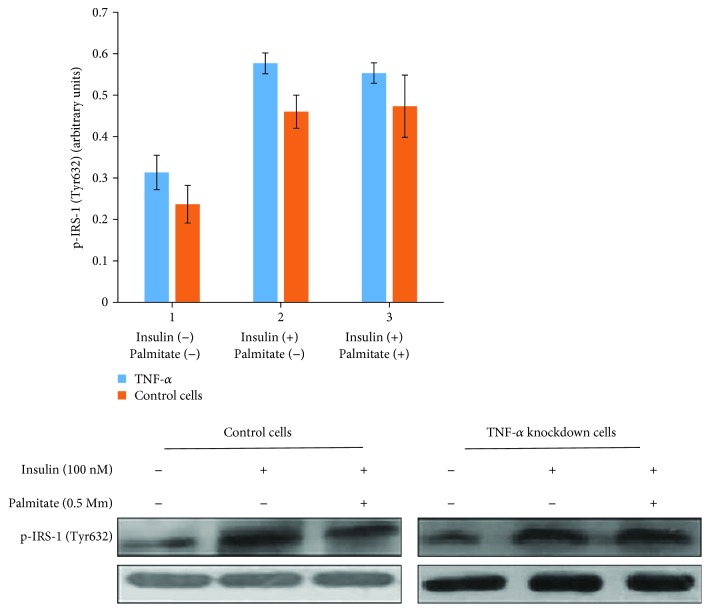
Effect of palmitate on the amount of Tyr632 phosphorylation of IRS-1 molecule in HepG2 cells and TNF-*α*-downregulated and normal cells. The cells were treated with palmitate and before harvesting was incubated in the presence and absence of insulin conditions. Western blot test was performed by using antibody against phosphotyrosine 632 of IRS-1 and IRS-1 (as internal control). The phosphorylation rate of tyrosine 632 of IRS-1 normalized with adjacent IRS-1 control molecules. The data has been achieved of three independent experiments and demonstrated as mean ± SD; *p* < 0.05.

To evaluate the insulin signaling, the positive phosphorylation of the key element in this pathway, IRS-1 (tyrosine 632), was analyzed by a Western blot method. In the absence of palmitate and no treatment with insulin, there was no any significant difference in tyrosine 632 phosphorylation of IRS-1 molecules in TNF-*α*-downregulated and normal cells (*p* < 0.05). The results showed the phosphorylation of tyrosine 632 of IRS-1 after treatment of cells with palmitate (to make the diabetic model of insulin resistance) and stimulation with insulin (to initiate the signal pathway) in TNF-*α*-downregulated cells was 45% (*p* < 0.05) higher than the normal cells. Following the absence of palmitate and stimulation with 100 nM insulin, the phosphorylation of IRS-1 in TNF-*α*-downregulated cells evaluated was 29% higher (*p* < 0.05) compared to the normal cells (Figure 3).

## 4. Discussion

Several genetic and environmental factors have been determined to involve in insulin resistance, of which TNF-*α* has taken much concentration recently. Studies have shown that insulin resistance is associated with elevated plasma levels of TNF-*α* [17] and causes a higher level of expression in tissues such as adipose [9, 10, 12] and skeletal muscle [8, 14, 18]. On the other hand, it has been frequently reported that the elevated level of saturated fatty acids, namely palmitate as the most abundant one in plasma, leads to insulin resistance in insulin target tissues [19–23]. In order to understand the possible role of TNF-*α* as an inflammatory mediator cytokine on insulin signaling inhibition, we designed the current course of TNF-*α* levels in the simulated obese HepG2 cells under treatment with palmitate. The idea is consistent with the previous studies regarding elevated expression of TNF-*α* in the liver [6, 7] and skeletal muscle in palmitate-induced insulin resistance condition [14, 15, 24–26]. Our results demonstrated the highest induced TNF-*α* protein levels on 16-hour treatment with palmitate (0.5 mM). This indicates the etiological role of elevated palmitate on TNF-*α* overexpression in obese insulin resistance hepatocytes. The molecular mechanisms of insulin resistance have been demonstrated extensively [27]. Multiple systemic factors associated with insulin resistance, for example, free fatty acids and TNF-*α*, can reduce IRS-1 serine phosphorylation and thus inhibits its function [28, 29]. Decreased IRS-1 serine phosphorylation is associated with negative effects on the insulin signaling pathway and has been described in connection with TNF-*α*-induced insulin resistance. The elevated TNF-*α* levels have been reported in obesity and other insulin-resistant states [30]. The experiment of TNF-*α* knockdown was carried out by shRNA-TNF-*α* lentiviral particles containing shRNA specifically for TNF-*α*. This process resulted in nearly 53.0% reduction in TNF-*α* protein levels (compared to normal cells). It is noted that due to *t*-test, the value rate of gene expression was reduced to *p* < 0.05 and considered to be significant. It suggests that the particles containing shRNA to silence TNF-*α* gene cause the efficient rate of TNF-*α* knockdown. The process of TNF-*α* downregulation was then followed by palmitate treatment to avoid coincidence reactions between TNF-*α* level changes and inhibition of insulin signaling. The tyrosine 632 phosphorylation of IRS-1 after treatment of cells with palmitate and stimulation with insulin showed less reduction of 55.0% (*p* < 0.05) in TNF-*α*-downregulated cells compared to the normal cells of 71.0% (*p* < 0.05). Although the treatment with palmitate 0.5 mM generally reduced the tyrosine 632 phosphorylation of the molecule IRS-1 in both TNF-*α*-downregulated and normal cells, the amount of phosphorylation in TNF-*α*-downregulated cells is approximately 1.3 times (*p* < 0.05) higher than the normal cells. The achieved results confirm that the reduction of TNF-*α* expression in HepG2 cells improves insulin-stimulated Tyr632 IRS-1 phosphorylation. Our results were parallel to the other studies approaching the reduction of TNF-*α* through different strategies and have demonstrated improved insulin sensitivity in skeletal muscle, liver cells, and animal models [6, 7, 15, 31–33]. To sum up, according to our data, palmitate can dysregulate the insulin signaling pathway and causes diabetic insulin resistance by increased expression of TNF-*α* in hepatocytes.

## 5. Conclusion

These data support the evidence that TNF-*α* downregulation contributes to the improvement of insulin sensitivity in hepatic cells, even in the presence of palmitate. Taking these findings together, the inhibition of TNF-*α* signaling has a potential possibility for treatment target. It could be a strong point in management of metabolic and cardiovascular diseases, lipid-induced insulin resistance, and type 2 diabetes.

## Data Availability

The data used to support the findings of this study are available from the corresponding author upon request.
